# Procalcitonin Kinetics in the First 72 Hours Predicts 30-Day Mortality in Severely Ill Septic Patients Admitted to an Intermediate Care Unit

**DOI:** 10.14740/jocmr2251w

**Published:** 2015-07-24

**Authors:** Filippo Pieralli, Vieri Vannucchi, Antonio Mancini, Elisa Antonielli, Fabio Luise, Lucia Sammicheli, Valerio Turchi, Ombretta Para, Francesca Bacci, Carlo Nozzoli

**Affiliations:** a Internal and Emergency Medicine Unit, Careggi University Hospital, Florence, Italy

**Keywords:** Procalcitonin, Sepsis, Biomarker, High dependency unit, Intermediate care unit

## Abstract

**Background:**

Severe sepsis and septic shock are leading causes of morbidity and mortality among critically ill patients, thus the identification of prognostic factors is crucial to determine their outcome. In this study, we explored the value of procalcitonin (PCT) variation in predicting 30-day mortality in patients with sepsis admitted to an intermediate care unit.

**Methods:**

This prospective observational study enrolled 789 consecutive patients with severe sepsis and septic shock admitted to a medical intermediate care unit between November 2012 and February 2014. Kinetics of PCT expressed as percentage were defined by the variation between admission and 72 hours, and 24 and 72 hours; they were defined as Δ-PCT0-72h and Δ-PCT24-72h, respectively.

**Results:**

The final study group of 144 patients featured a mean age of 73 ± 14 years, with a high prevalence of comorbidities (Charlson index greater than 6 in 39%). Overall, 30-day mortality was 28.5% (41/144 patients). A receiver-operating-characteristic (ROC) analysis identified a decrease of Δ-PCT0-72h less than 15% (area under the curve: 0.75; 95% confidence interval (CI): 0.67 - 0.82) and a decrease of Δ-PCT24-72h less than 20% (area under the curve: 0.83; 95% CI: 0.74 - 0.92) as the most accurate cut-offs in predicting mortality. Decreases of Δ-PCT0-72h less than 15% (HR: 3.9, 95% CI: 1.6 - 9.5; P < 0.0001) and Δ-PCT24-72h less than 20% (HR: 3.1, 95% CI: 1.2 - 7.9; P < 0.001) were independent predictors of 30-day mortality.

**Conclusions:**

Evaluation of PCT kinetics over the first 72 hours is a useful tool for predicting 30-day mortality in patients with severe sepsis and septic shock admitted to an intermediate care unit.

## Introduction

In recent years, an increasing number of studies have confirmed procalcitonin (PCT) to be one of the most reliable serological markers for bacterial infection and sepsis [1-3]; in particular, its use has become widespread (first in Europe and, recently, in the US since its approval by the FDA in 2006) for differentiating between sepsis and systemic inflammatory response syndrome (SIRS) [3], and guiding empirical antibiotic therapy [4, 5] in community-acquired pneumonia and sepsis.

PCT is a 116-amino acid prohormone of the calcium metabolism regulator, calcitonin that is primarily expressed in the thyroid and in extra-thyroidal tissues (neuroendocrine tissue) in response to lipopolysaccharides and bacterially induced cytokines. Being released by parenchymal cells, including the liver cells, adipocytes, and muscle cells [2], plasma PCT levels start to rise within 2 - 6 h of initial clinical manifestation of sepsis, and fall if the septic process is controlled. Conversely, increase of PCT is down-regulated [6] in patients with viral infections, who experience a very low or negligible increase in PCT levels [1].

Although there is still some debate on the exact mechanism of PCT and its diagnostic and prognostic potential and limitations, PCT is currently accepted as the serological marker of reference for infections and outcome for critically ill patients [1, 2, 7, 8].

So far, however, most trials in literature have tested the effectiveness of PCT exclusively on patients within the intensive care unit (ICU) and emergency department (ED), without extending findings to other patient settings, such as that of the intermediate care units (IMC), which feature peculiar demographic and clinical characteristics. Differently from the heterogeneous group of ICU and ED patients, who present a wide range of sever trauma/injury or acute conditions across all age groups, IMC patients are generally elderly patients, who present several comorbidities and chronic conditions. Such patients require a more intensive monitoring and assistance than that provided by general medical wards, but yet do not require life-support equipment/assistance (such as invasive mechanical ventilation) as in the ICU or ED. In fact, IMCs, also known as step-down units or high-dependency units [9, 10], admit both patients from the ICU who have been stabilized, and patients from the emergency room and general medical wards who are at higher potential risk of complications [9, 11].

In the case of patients with sepsis or septic shock, the availability of reliable prognostic markers becomes fundamental in supporting quick decision-making and delivering most appropriate and timely care to most critical patients.

Here we report a prospective observational study that aimed to explore the role of PCT and its variations between admission and 72 h in predicting 30-day mortality in patients with severe sepsis and septic shock admitted to a medical IMC.

## Patients and Methods

### Study design, patients, and clinical setting

This was a prospective non-interventional observational study performed at the IMC within the Emergency and Internal Medicine Department of the Careggi Hospital in Florence (Italy).

The study considered 789 consecutive patients admitted to our unit, between November 2012 and February 2014, enrolling a total 149 patients presenting sever sepsis or septic shock, as defined by the American College of Chest Physicians/Society of Critical Care Medicine Consensus Conference [12]. Patients had been admitted directly from the ED or from general medical wards within 12 h from onset of sepsis, in accordance with the internationally accepted criteria for admission to IMC [11]. Patients transferred by step-down from ICU were not included in the study.

The only criterion for exclusion from the study was an incomplete profile of predetermined PCT measurements within 72 h since admission.

The study protocol was submitted and approved by our Local Ethics Committee and the study was performed in compliance with the principles set in the Declaration of Helsinki.

### Patient management and outcome measures

Patients were admitted to our IMC, an eight-bed section within the Emergency and Internal Medicine Department, where continual patient-care is guaranteed on rotating basis by five dedicated internal medicine specialists trained in critical care medicine (covering daily shifts) who were supported by five non-dedicated internal medicine physicians for the coverage of night shifts and nursing staff distributed by a 1:4 ratio, and who are assisted by one nurse assistant during day shifts.

As foreseen by our unit’s patient management protocol, patients with most severe cases of sepsis (i.e. severe sepsis and septic shock) underwent routine continuous monitoring for ECG, oximetry, respiratory rate, arterial blood pressure (non-invasive or invasive, as needed), central venous pressure (Infinity Delta, Drager, Lubeck, Germany) and regular monitoring of central venous saturation of oxygen (ScVO_2_) and lactate values. Non-invasive mechanical ventilation was provided only when required. As established by the guidelines of the Surviving Sepsis Campaign [12], multiple blood and urine sample cultures were also performed (BACTEC system, Benex Limited, Shannon, Ireland). Cultures from sputum, bronchial aspirate or purulent exudates were obtained in selected cases.

Empiric antibiotic therapy was given early and later modified if necessary, based on results of cultural samples. After a first-line evaluation with a chest X-ray, abdominal ultrasound and transthoracic echocardiography, a complete thoracic and abdominal CT scan was performed. Severity of condition was classified according to three different score-systems: the mortality in emergency department sepsis (MEDS), sepsis-related organ failure assessment (SOFA), acute physiology and chronic health evaluation (APACHE) II score [13-15], plus the burden of multimorbidities with the age-adjusted Charlson comorbidity index [16]. Sepsis was considered nosocomial when onset occurred more than 2 days after hospital admission. The clinical end-point of the study was 30-day mortality.

### Determination of procalcitonin

Blood samples were drawn for measurement of PCT within the first hour after admission to the IMC, and again at 24 and 72 h. PCT was measured using the Vidas PCT kit (Brahms Diagnostics, Berlin, Germany) in accordance to manufacturer’s instructions and as described elsewhere [17].

### Statistical analysis

Data were expressed as mean ± standard deviation or as proportions. Student’s *t*-test was used for the comparison of normally distributed continuous data, and the Fisher’s exact test for the comparison of non-continuous variables. Kinetics of PCT were defined by the variation of values between admission and 72 h, and 24 and 72 h. They were calculated by the ratio of the difference between the second and the first measurement divided by the first measurement, expressed as percentage (e.g. (value at 72 h - value on admission)/value on admission per 100]. These variables were then defined as Δ-PCT0-72h and Δ-PCT24-72h, respectively. A receiver-operating-characteristic (ROC) analysis was used to obtain the most accurate cut-off of single Δ values for the identification of the clinical end-point. Relative hazard (HR) of variables and 95% confidence intervals (CIs) were calculated using univariate and multivariate logistic regression analysis. Multivariate analysis was performed using a stepwise forward regression model, with an entry probability for each variable set at 0.05. Survival curves were constructed according to the Kaplan-Meier method. All P-values were two-tailed and considered significant when < 0.05 (95% CI). All analyses were performed using 21.0 SPSS statistical software (SPSS, Chicago, IL).

## Results

### Patient characteristics and outcome

Between November 2012 and February 2014, 789 patients were admitted to our IMC. Of the 149 (19%) patients presenting severe sepsis or septic shock, five were excluded from the study: two due to incomplete/unavailable profile of predetermined PCT measurements, and three patients because of death or transfer to the ICU within 72 h from admission. Hence the final study population was composed of 144 patients.

As shown in Table 1 summarizing main demographic and clinical characteristics at baseline, the patient population was mostly composed of older individuals and had a high prevalence of comorbidities, with over 35% of the patients having two or more significant pre-existing diseases. Of note, a relevant proportion of patients had dementia and/or chronic total dependence. Thirty-nine percent of the population presented an age-adjusted Charlson comorbidity index > 6. Patients with poorest outcome had the highest baseline severity of disease, independently from the scoring system used. Values of general laboratory parameters did not differ between patients who deceased within the 30 days end-point and those who survived. The most common site of infection was the lung (51.4%) and most infections were community acquired (77%) (Table 1). Ninety-three patients (64.6%) had severe sepsis upon admission, while the remaining had septic shock (35.4%). Blood cultures were positive in nearly 46% of cases, with a prevalence of Gram-negative bacteria. There was a significant prevalence of patients with multiple drug-resistant (MDR) bacterial infections (methicillin-resistant *S. aureus, P. aeruginosa, A. baumannii, K. pneumoniae* and *S. maltophilia*) accounting for 17.4% of isolated agents, with no difference between groups, nor significance at univariate analysis for prediction of mortality (HR: 2.4; 95% CI: 0.9 - 6.3; P = 0.08).

**Table 1:** Baseline Characteristics of the Overall Population and in the Two Groups of Patients Who Survived or Died at 30-Day Follow-Up

	Overall population (n = 144)	Death at 30 days		P
		No (n = 103)	Yes (n = 41)	
Age (years)	73 ± 14	72.3 ± 14.7	74.9 ± 12.9	0.32
Male	50 (34.7%)	37 (35.9%)	13 (31.7%)	0.70
Comorbidities				
CHF (NYHA class III-IV)	35 (24.3%)	25 (24.3%)	10 (24.4%)	0.99
COPD	29 (20.1%)	21 (20.4%)	8 (19.5%)	0.99
Diabetes	30 (20.8%)	23 (22.3%)	7 (17.1%)	0.65
Chronic renal failure	26 (18.1%)	17 (16.5%)	9 (22.0%)	0.47
Active cancer	23 (16%)	13 (12.6%)	10 (24.4%)	0.12
Hematologic malignancy	18 (12.5%)	10 (9.7%)	8 (19.5%)	0.16
Chronic steroid therapy	27 (24.1%)	15 (18.8%)	12 (37.5%)	0.05
Age-adjusted Charlson comorbidity index	5.9 ± 2.3	5.7 ± 2.4	6.5 ± 1.8	0.07
Chronic total dependence	42 (29.2%)	29 (28.1%)	13 (31.7%)	0.40
Clinical parameters				
Heart rate (bpm)	97 ± 20	96 ± 19	99 ± 20	0.42
Respiratory rate (bpm)	19.2 ± 6.6	18.6 ± 6.7	20.7 ± 6.4	0.82
MAP (mm Hg)	83.1 ± 15.5	83.2 ± 16.5	82.8 ± 13.0	0.89
Temperature (°C)	37.2 ± 1.0	37.2 ± 1.0	37.1 ± 0.9	0.34
PaO_2_/FiO_2_ (mm Hg)	243.8 ± 94.2	268.7 ± 85.6	192.2 ± 91.5	< 0.001
Indexes of severity				
APACHE-II (points)	15.8 ± 5.9	14.9 ± 5.7	17.9 ± 6.2	< 0.05
MEDS (points)	9.3 ± 4.5	8.2 ± 4.1	12.2 ± 3.9	< 0.0001
SOFA (points)	5.7 ± 3.5	4.9 ± 3.3	7.6 ± 3.2	< 0.0001
Length of stay (days)	14.6 ± 11.2	15.7 ± 11.6	11.9 ± 9.9	0.52

CHF: chronic heart failure; COPD: chronic obstructive pulmonary disease; MAP: mean arterial pressure; APACHE: Acute Physiology, Age and Chronic Health Evaluation; MEDS: mortality in emergency department sepsis score; SOFA: sepsis-related organ failure assessment score.

Overall, death within 30 days from the day of admission occurred in 41 patients (28.5%); patients admitted with septic shock had a 35.4% mortality rate. On average, death occurred 14 ± 12 days from admission to the IMC (range 3 - 28 days).

### Biohumoral and clinical predictors of outcome

**Figure 1:**
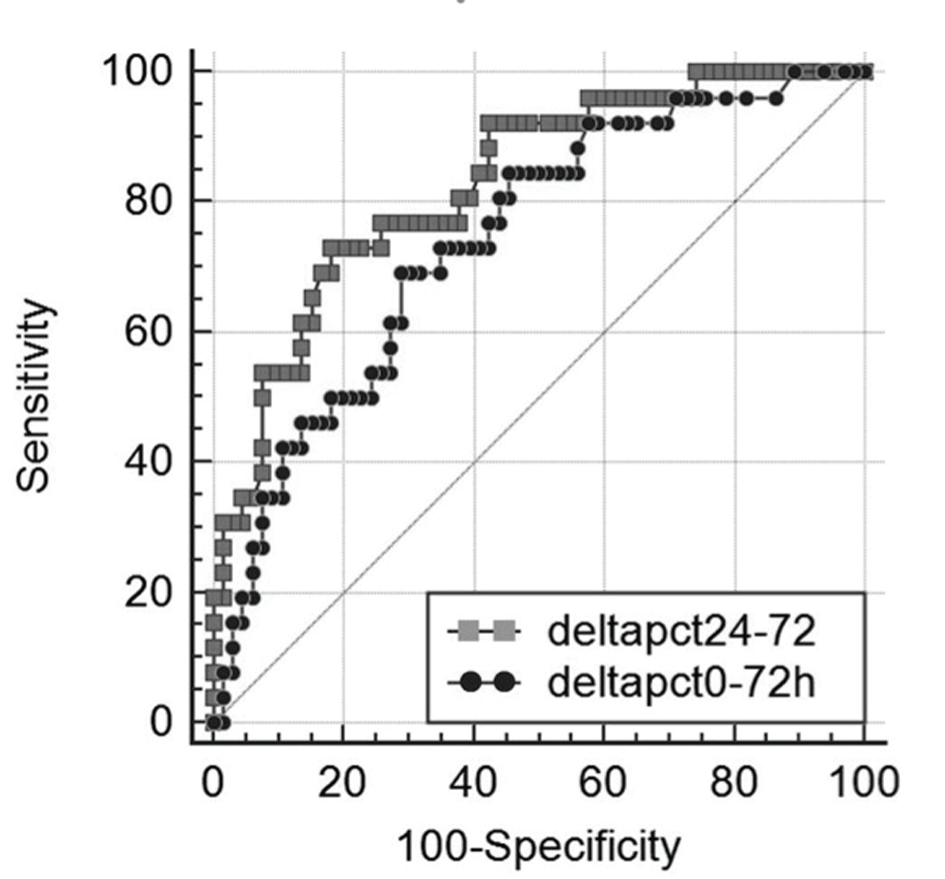
Receiver operating characteristic (ROC) curves of Δ-PCT% variation between day 0 and 72 h (black dotted line) and between 24 and 72 h (grey dotted line) for differentiating between 30-day survivors and non-survivors in 144 patients with severe sepsis syndromes, area under the ROC curve = 0.743 (0.055) for and 0.83 (0.046) (mean (standard deviation)), respectively (P = 0.052).

Mean PCT values on admission were 28.06 ± 63.29 ng/mL (range 0.05 - 397.92 ng/mL) and were lower in patients who died within the 30-day timeframe (Table 2). A significant decrease of mean PCT values between admission and 72 h was found in survivors (P < 0.001; 95% CI: 8.9 - 32.7) as compared to those who died (P = 0.665; 95% CI: -13.1 - 10.8). An ROC analysis identified a decrease of Δ-PCT0-72h less than 15% (area under the curve: 0.75; 95% CI: 0.67 - 0.82) and a decrease of Δ-PCT24-72h less than 20% (area under the curve: 0.83; 95% CI: 0.74 - 0.92) as the more accurate cut-offs in predicting adverse outcome. The predictive value of Δ-PCT0-72h was compared with Δ-PCT24-72h through construction of the corresponding ROC curves; areas under the curve were 0.74 (0.05) and 0.83 (0.04) (mean (standard deviation)), respectively (P = 0.052) (Fig. 1).

**Table 2:** Baseline Laboratory Characteristics, Site and Type of Infection, and Isolated Microbiological Agent in the General Population and in the Groups of Patients Who Survived or Died at 30-Day Follow-Up

	Overall population (n = 144)	Death at 30 days		P
		No (n = 103)	Yes (n = 41)	
Laboratory values				
WBC count (1,000/mm^3^)	10.4 ± 9.3	10.9 ± 8.4	11.1 ± 11.3	0.54
Hemoglobin (g/dL)	10.7 ± 1.7	11.1 ± 2.2	10.7 ± 1.9	0.29
Platelet count (1,000/mm^3^)	210 ± 143	204 ± 136	223 ± 159	0.47
Creatinine (mg/dL)	1.7 ± 1.8	1.9 ± 1.7	1.3 ± 0.7	0.67
Lactate (mmol/L)	2.3 ± 1.6	2.4 ± 1.9	2.1 ± 1.0	0.38
Cardiac troponin I (μg/L)	0.3 ± 0.5	0.3 ± 0.6	0.1 ± 0.3	0.21
ProBNP (pg/mL)	9,244 ± 14,805	9,613 ± 16,604	7,998 ± 7,301	0.66
PCT on admission (ng/mL)	28.06 ± 63.29	34.1 ± 70.1	12.7 ± 33.7	0.07
PCT at 24 h (ng/mL)	29.09 ± 63.28	33.64 ± 70.05	16.81 ± 37.87	0.19
PCT at 72 h (ng/mL)	13.48 ± 29.04	13.31 ± 31.97	13.92 ± 20.21	0.91
Site and type of infection				
Lung	74 (51.4%)	47 (45.6%)	27 (65.9%)	0.04
Urinary tract	32 (22.2%)	30 (29.1%)	2 (4.9%)	0.001
Gall bladder	6 (4.2%)	5 (4.9%)	1 (2.4%)	0.67
Other	32 (22.2%)	21 (20.4%)	11 (26.8%)	0.69
Septic shock	51 (35.4%)	25 (24.3%)	26 (63.4%)	< 0.0001
Nosocomial	33 (22.9%)	21 (20.4%)	12 (29.3%)	0.27
Community acquired	111 (77.1%)	82 (79.6%)	29 (70.7%)	0.27
Isolated agent				
Positive blood cultures	67 (46.5%)	49 (47.6%)	18 (43.9%)	0.42
Gram-positive	27 (18.7%)	17 (16.5%)	10 (24.4%)	0.26
Gram-negative	30 (20.8%)	24 (23.3%)	6 (14.6%)	0.15
Fungi	3 (2.1%)	2 (1.9%)	1 (2.4%)	0.77
Polymicrobial	7 (4.9%)	11 (10.7%)	6 (14.6%)	0.63

WBC: white blood cell count; ProBNP: pro-brain natriuretic peptide; PCT: procalcitonin.

**Figure 2:**
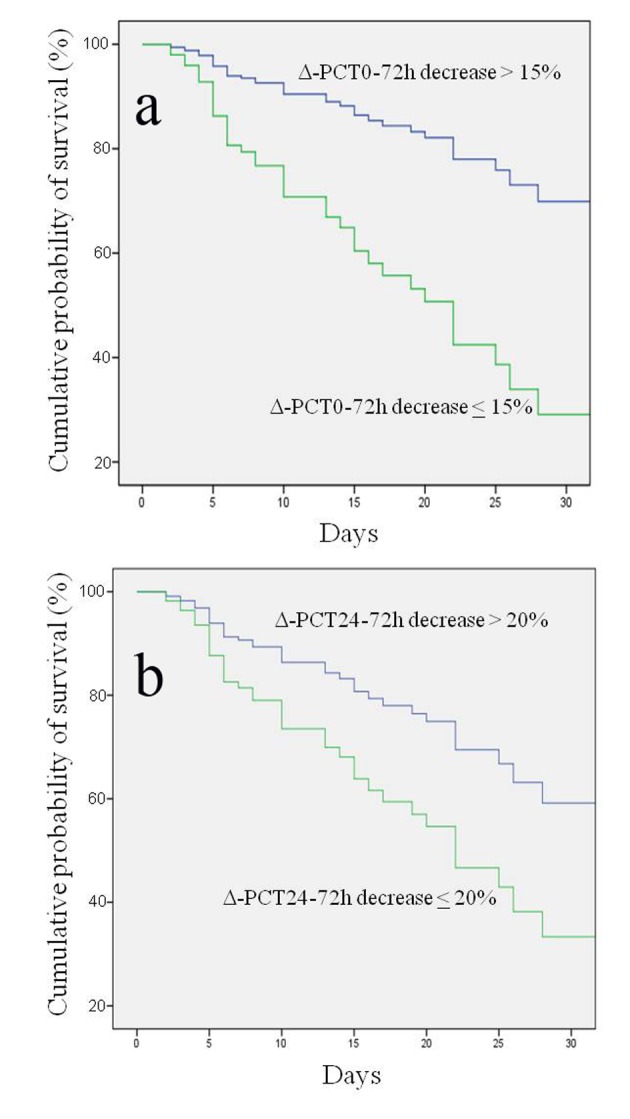
Kaplan-Meier estimation of probability of survival curves for different procalcitonin percentage variations (Δ-PCT) at different time intervals. (a) Δ-PCT percentage decrease between day 0 and 72 h greater or less than 15% in predicting survival; log rank test P < 0.000; 95% CI: 29.8 - 41.3. (b) Δ-PCT percentage decrease between 24 and 72 h greater or less than 20% in predicting survival; log rank test P < 0.0001; 95% CI: 11.4 - 27.8.

Results of univariate and multivariate logistic regression analysis of several demographic, clinical characteristics and biohumoral markers analysed are shown in Table 2. Interestingly, the univariate logistic regression analysis highlighted that a decrease of Δ-PCT0-72h < 15% was predictive of adverse outcome with a 6.1 HR (95% CI: 2.7 - 13.6; P < 0.0001) and that a Δ-PCT24-72h < 20% was predictive of adverse outcome with a 5.9 HR (95% CI: 2.5 - 14.1; P < 0.0001). At multivariate logistic regression analysis assessing the association of several variables with risk of 30-day mortality, Δ-PCT0-72h decrease less than 15% and Δ-PCT24-72h decrease less than 20% retained their independent predictive role with an HR of 3.9 (95% CI: 1.6 - 9.5; P < 0.0001) and of 3.1 (95% CI: 1.2 - 7.9; P < 0.001), respectively (Table 3). Kaplan-Meier event-free survival estimate curves for the corresponding Δ-PCT values are shown in Figure 2.

**Table 3:** Factors Predictive of In-Hospital Death at Univariate and Multivariate Logistic Regression Analysis

	Univariate analysis			Multivariate analysis		
	HR	95% CI	P	HR	95% CI	P
Age	1.0	0.9 - 1.0	0.32			
Age more than 75 years	1.0	0.5 - 2.1	0.98			
Sex (male vs. female)	0.8	0.4 - 1.8	0.70			
Diabetes	0.7	0.3 - 1.8	0.48			
Chronic heart failure	1.0	0.4 - 2.3	0.98			
COPD	0.9	0.4 - 2.3	0.90			
Chronic renal failure	1.4	0.6 - 3.5	0.44			
Cancer	2.2	0.9 - 5.6	0.87			
Hematologic malignancy	2.2	0.8 - 6.2	0.11			
Chronic total dependence	1.2	0.5 - 2.6	0.77			
Corticosteroid therapy	2.6	1.0 - 6.4	0.04			
Charlson index*	1.2	1.0 - 1.4	0.08			
Pneumonia	2.3	1.1 - 4.9	0.03			
Polymicrobial sepsis	2.5	1.1 - 5.9	< 0.05			
MDR bacteria	2.4	0.9 - 6.3	0.08			
Nosocomial acquired	1.6	0.7 - 3.7	0.27			
Septic shock on presentation	5.4	2.5 - 11.8	< 0.001	5.3	2.3 - 12.4	< 0.0001
Antibiotic therapy within 6 h	0.4	0.1 - 1.3	0.19			
MEDS	1.3	1.1 - 1.4	< 0.001	1.3	1.1 - 1.5	0.001
SOFA	1.3	1.1 - 1.4	< 0.001			
SOFA 48 h	1.3	1.1 - 1.6	< 0.001			
APACHE-II	1.1	1.0 - 1.1	0.02			
PCT on admission	0.9	0.9 - 1.0	0.09			
PCT at 24 h	0.9	0.9 - 1.0	0.22			
PCT at 72 h	1.0	0.9 - 1.0	0.90			
Δ-PCT % variation						
Δ-0-72h decrease < 15%	6.1	2.7 - 13.6	< 0.0001	3.9	1.6 - 9.5	< 0.0001
Δ-24-72h decrease < 20%	5.9	2.5 - 14.1	< 0.0001	3.1	1.2 - 7.9	< 0.001

*Age-adjusted Charlson comorbidity index [19]. COPD: chronic obstructive pulmonary disease; MDR: multiple drug-resistant; MAP: mean arterial pressure; MEDS: mortality in emergency department sepsis; SOFA: sepsis-related organ failure assessment; APACHE: acute physiology, age and chronic health evaluation; PCT: procalcitonin. Δ-PCT% variation is the ratio, expressed as percentage, of the difference between the second and the first measurement divided by the first measurement (see “Methods” section for more details).

## Discussion

While many studies have validated the use of PCT for risk stratification in the ICU and the ED [18-24], the present study is the first to investigate the potential role of PCT in risk stratification on a large cohort of patients presenting severe sepsis or septic shock within the IMC setting. This aspect is particularly relevant in the European context where ICMs have a central role in relieving the ICU from patient overflow; indeed, compared to other healthcare realities such as the United States, the number of ICU beds in most facilities is extremely limited compared to the patient population they are meant to serve [25, 26]. Several studies have demonstrated the convenience of implementing IMCs in support to ICUs [9], and IMCs are now slowly being established in many hospital facilities with the specific purpose of assisting/caring severely ill patients with limited organ dysfunction, advanced age, or a heavy burden of comorbidities. Hence it is important that parameters which are currently validated for other settings be reconsidered in view of this new growing hospital population.

In the present study, one major finding emerging is the identification of independent predictors of 30-day mortality, namely 1) a PCT decrease less than 15% between admission and 72 h and 2) a PCT decrease less than 20% between 24 and 72 h from onset. Such results were independent of initial severity assessment by commonly used risk score, such as APACHE II, SOFA and MEDS. At multivariate analysis patients with a decline in Δ-PCT0-72h less than 15% had a 3.9-fold risk increase of 30-day mortality and those with a decline in Δ-PCT24-72h less than 20% had a 3.1-fold increase. Conversely, absolute concentrations of PCT at baseline, and at subsequent time intervals over 72 h, had no role in predicting 30-day mortality. Therefore, dynamic changes of PCT, rather than absolute concentrations, were predictive of adverse outcome. Our results are in agreement with those reported by Phua et al who described no value of initial PCT determination in differentiating survivors and non-survivors in a population of ICU patients with septic shock. Only a rising trend in PCT values between day 1 and 2 was predictive of 28-day mortality [27]. Similarly, in septic shock patients with initial PCT values greater than 10 ng/mL, Guan et al reported that dynamic change, instead of PCT concentrations itself, was predictive of mortality [28]. Charles et al found that a decrease of PCT greater of more than 30% between day 2 and 3 was predictive of a better outcome in a cohort of septic patients admitted to ICU [5]. More recently Schuetz et al in a retrospective study of patients with severe sepsis or septic shock admitted to US critical care units found that 72-h PCT kinetics was an accurate predictor of in-hospital mortality [29, 30]. In our study, we did not find any significant difference in PCT kinetics considered at two different time intervals; indeed there was no difference between area under the ROC curves of Δ-PCT0-72h and Δ-PCT24-72h (0.74 and 0.83, respectively; P = 0.052). The superiority of kinetics rather than absolute PCT values in prognostication seems to rely on PCT physiological characteristics. There is evidence that there is little intracellular PCT storage and its production in response to bacterial infections is mostly mediated by activation of the common ancestral calcitonin gene I in parenchymal tissues [30]; this process needs time and could be the reason of the greater prognostic value of repeated PCT measurements exploring dynamic variations instead of absolute concentrations.

There are some characteristics in our cohort of patients that should be underlined. In comparison to previous studies on prognostic value of PCT in severe sepsis syndromes, our patients were significantly older and the mean age was nearly one decade higher (73 vs. 64 years) than that reported in other studies [5, 24, 27-29]. Moreover, the prevalence of comorbid conditions was high as documented by age adjusted Charlson comorbidity index greater than 6, corresponding to an estimated 1-year mortality of 63%, in nearly 40% of the study population [16]. The 30-day mortality rate in our cohort was 28.5%, a value comparable to recent data regarding severely ill septic patients admitted in European ICUs [31]. The advanced age and the burden of comorbidity could explain this finding despite a lower risk profile on admission evaluated with commonly used risk scores in sepsis (i.e. APACHE II, SOFA, MEDS) [15, 31-33]. These indexes of severity may be less reliable in a setting different from that of the ICU or ED for which they were initially developed and validated [34]. Thus, biohumoral markers, such as PCT, may have a more accurate predictive role in the setting of IMCs than risk scores [35].

Although promising, our results must however be viewed in light of some limitations within the study, such as the single center experience and the arbitrary choice of percentage changes as an indicator of PCT kinetics instead of other methods. Some authors for the purpose of similar studies considered PCT percentage changes grouped in tertiles or quartiles, while others reported specific cut-off values for PCT interpretation. Certainly, choosing in favor a specific method over another has advantages and disadvantages. For example, dividing percentage variation into tertiles or quartiles can help to understand the impact on mortality across different degrees of change, but can be more difficult to export. Moreover, dynamic changes expressed as percentage variation depend on many factors such as the starting value, and subsequent timing of PCT evaluation, on the other hand they can be easily quantified and have been used in many papers. A direct comparison of results of studies is difficult for several reasons; one among others is that all the trials on the prognostic role of biomarkers in sepsis were performed in ICU or ED and there are no available data in intermediate care setting. Furthermore, a possible limitation of the study is the exclusion of patients where PCT measurements were not available for several reasons. In our opinion this is a minor limitation since these patients were few (3.3% of screened patients) and the study sought to evaluate PCT kinetics between admission and 72 h “per protocol”.

In summary, our results show that a PCT decrease between admission and 72 h less than 15%, and between 24 and 72 h less than 20%, are independent predictors of 30-day mortality in severely ill septic patients admitted to a hospitalist-managed IMC. Therefore, in the broader context of other routine clinical and laboratory parameters, evaluation of PCT kinetics over the first 72 h is a useful tool for adverse outcome prognostication in patients with severe sepsis and septic shock admitted to an IMC. Importantly, this provides physicians additional aid in the decision-making process for care intensification and proper allocation of hospital resources.
